# The Diagnosis of Nontuberculous Mycobacterial Pulmonary Disease by Single Bacterial Isolation Plus Anti-GPL-Core IgA Antibody

**DOI:** 10.1128/spectrum.01406-21

**Published:** 2022-01-05

**Authors:** Takahiro Kawasaki, Seigo Kitada, Kiyoharu Fukushima, Eri Akiba, Kako Haduki, Haruko Saito, Tadayoshi Nitta, Akira Kawano, Akito Miyazaki, Takuro Nii, Tomoki Kuge, Taro Koba, Takanori Matsuki, Kazuyuki Tsujino, Keisuke Miki, Ryoji Maekura, Hiroshi Kida

**Affiliations:** a National Hospital Organization Osaka Toneyama Medical Center, Toyonaka, Osaka, Japan; b Department of Respiratory Medicine and Clinical Immunology, Osaka University Graduate School of Medicine, Suita, Osaka, Japan; c Kitada Respiratory Clinic, Yao, Osaka, Japan; d Graduate School of Health Care Sciences, Jikei Institute, Osaka, Japan; University of Mississippi Medical Center

**Keywords:** anti-glycopeptidolipid-core IgA antibody, single isolation, nontuberculous mycobacterial pulmonary disease, *Mycobacterium avium* complex, *Mycobacterium abscessus*, *Mycobacteroides abscessus* complex, contamination, diagnosis

## Abstract

Although serum anti-glycopeptidolipid (GPL)-core IgA antibody is a highly specific test for infection with Mycobacterium avium complex (MAC), Mycobacterium abscessus, and its subspecies *abscessus*, subsp. *massiliense*, and subsp. *bolletii* (MAB), its use for the definitive diagnosis of MAC pulmonary disease (PD) and MAB-PD are unknown. To clarify the diagnostic accuracy of the anti-GPL-core IgA antibody test among patients with radiologically suspected MAC-PD or MAB-PD who already have a single positive sputum culture test. The first isolations of MAC and MAB from patients with radiologically suspected MAC-PD or MAB-PD at the Osaka Toneyama Medical Center between January 2006 and December 2020 were collected. Patients were enrolled when their serum anti-GPL-core IgA antibody was measured during the 3 months before and after the first isolation. We retrospectively compared the results of anti-GPL-core IgA antibody testing with the final diagnoses based on the current guidelines. We included 976 patients for analysis. The serum anti-GPL-core IgA antibody was positive in 699 patients (71.6%). The positive predictive value of anti-GPL-core IgA antibody for the diagnosis of MAC-PD or MAB-PD was 97.4%. The median time required for the second positive culture after the first isolation was 51 days (interquartile range 12 to 196 days). The positive serum anti-GPL-core IgA antibody test allowed an early and definitive diagnosis of MAC-PD or MAB-PD in those who already had a single positive sputum culture test.

**IMPORTANCE** To satisfy the microbiologic criteria of the current diagnostic guideline for nontuberculous mycobacterial pulmonary disease (PD), at least two positive sputum cultures of the same species of mycobacteria from sputum are required to avoid the casual isolation of mycobacteria. This study showed that the positivity of a serum anti-glycopeptidolipid (GPL)-core IgA antibody test has an excellent diagnostic ability among patients with radiologically suspected Mycobacterium avium complex (MAC)-PD or Mycobacterium abscessus (MAB)-PD who already had a single positive sputum culture test. The usage of single culture isolation plus anti-GPL-core IgA antibody as another diagnostic criterion has a time, cost, and effort-saving effect. Furthermore, it will facilitate the diagnosis of MAC-PD or MAB-PD in the early stage of disease because serum anti-GPL-core IgA antibody becomes high in these patients. Therefore, we proposed adding single culture isolation plus anti-GPL-core IgA antibody as “combined microbiological and serological criteria” to the diagnostic guidelines for MAC-PD and MAB-PD.

## INTRODUCTION

The rate of nontuberculous mycobacterial pulmonary disease (NTM-PD) has increased recently worldwide (1, 2). Although NTM comprises approximately 200 species, Mycobacterium avium complex (MAC), represented by M. avium and M. intracellulare, and Mycobacterium abscessus and its subspecies *abscessus*, subsp. *massiliense*, and subsp. *bolletii* (MAB), are the major causative agents of NTM-PD in many countries, including Japan (3). In contrast to Mycobacterium tuberculosis, NTM exists in environmental sources, including water and soil (4). Therefore, the repeated isolation of the same species of mycobacteria from sputum is required for the diagnosis of NTM-PD, considering the possibility of contamination from the environment or colonization of mycobacteria in the respiratory tract (5).

The current American Thoracic Society/European Respiratory Society/European Society of Clinical Microbiology/Infectious Disease Society of America (ATS/ERS/ESCMID/IDSA) guidelines recommend the use of clinical, radiologic, and microbiologic criteria for the diagnosis of NTM-PD (6). This requires at least two positive sputum cultures of the same species of mycobacteria to satisfy the microbiologic criteria. This is based on a study that reported that 98% of patients with ≥2 sputum cultures had clinically significant MAC-PD (5). In that report, the authors stated that the probability of the casual isolation of the organism twice was low, and, therefore, a diagnosis of pulmonary infection caused by this organism could be made. However, a sputum culture is sometimes difficult and time-consuming for patients with early-stage disease who do not have sputum of appropriate quality or in hospitals that are unfamiliar with mycobacterial infectious diseases. Furthermore, waiting for a definitive MAC-PD diagnosis by two or more sputum cultures could lead to delays in appropriate disease management, in particular in patients with serious complications or under immunosuppressive therapy.

Anti-glycopeptidolipid (GPL)-core IgA antibody is a useful serological diagnostic tool for MAC-PD (7). GPL is a major cell wall component of NTM. MAC, MAB, Mycobacterium chelonae, Mycobacterium fortuitum, and Mycobacterium scrofulaceum contain GPL whereas M. tuberculosis and other NTM species, including Mycobacterium kansasii, do not (8). Levels of anti-GPL-core IgA antibody are specifically elevated in the sera of patients with MAC and MAB infections (7, 9, 10). Compared to other isotypes of antibody, anti-GPL-core IgA showed the best sensitivity and specificity for the diagnosis of MAC-PD (11). Although geographical differences might exist, it is rare for anti-GPL-core IgA to be casually elevated among patients without MAC or MAB infection (7, 10, 12–14). In addition, serum anti-GPL-core IgA is high in patients with early-stage MAC-PD (15).

We hypothesized that the probability of the casual elevation of serum anti-GPL-core IgA in patients with one or more isolation of mycobacteria is extremely low. In this study, we aimed to clarify the diagnostic accuracy of serum anti-GPL-core IgA antibody test among patients with radiologically suspected MAC-PD or MAB-PD who already have a single positive sputum culture test.

## RESULTS

**FIG 1:**
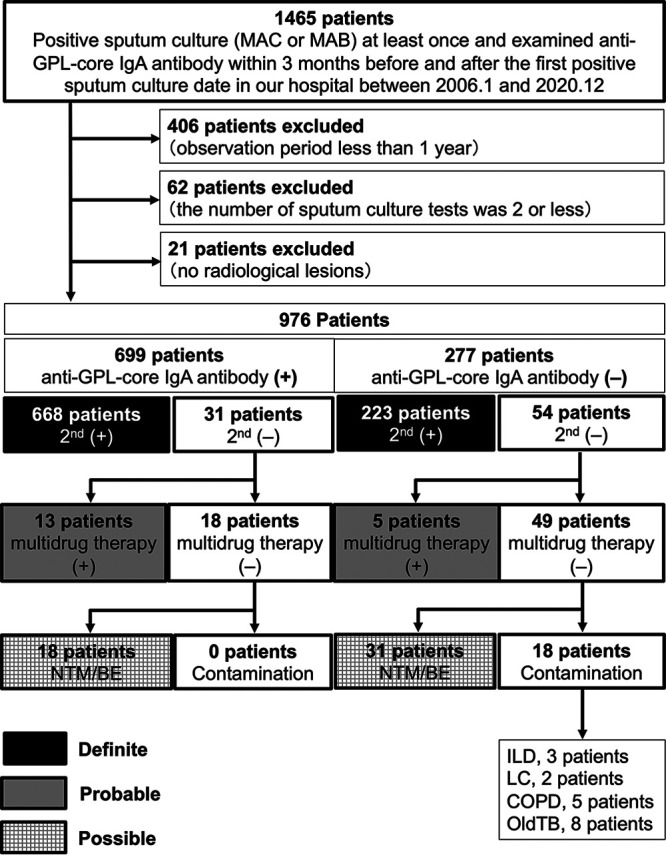
Flowchart of patient selection. Anti-GPL-core IgA antibody (+), positive anti-GPL-core IgA antibody (≥0.7 U/mL) at the first positive sputum culture; anti-GPL-core IgA antibody (−), negative anti-GPL-core IgA antibody (<0.7 U/mL) at the first positive sputum culture; 2nd (+), additional positive sputum culture isolation after the first positive sputum culture; 2nd (−), no positive sputum culture isolation after the first positive sputum culture; multidrug therapy (+), presence of history of taking multidrug therapy, including clarithromycin; multidrug therapy (−), no history of multidrug therapy, including clarithromycin; NTM/BE, patients with radiological pulmonary lesions compatible with NTM or bronchiectasis; contamination, patients with no obvious radiological pulmonary lesions of NTM or bronchiectasis; ILD, interstitial lung disease; LC, lung cancer; COPD, chronic obstructive pulmonary disease; old TB, past M. tuberculosis disease.

Overall, 1465 patients had at least one positive sputum culture test and were examined for serum anti-GPL-core IgA antibody within the 3 months before and after the sampling of the first positive sputum culture. We excluded 406 patients because of a short observation period (less than 1 year) and 62 patients because of an inadequate number of sputum culture tests (less than 3) to avoid the cases for which no clear conclusion can be drawn. We also excluded 21 patients because they had no radiological NTM lesion. The remaining 976 patients were included for analysis (Fig. 1). The baseline patient characteristics are shown in Table 1. Of these patients, 239 were male and 737 were female. Their ages were 70.5 ± 10.2 for males and 67.4 ± 11 years for females (mean ± standard deviation). The follow-up period after the first positive sputum culture test was 1518 [867 to 2594] days (median [interquartile range (IQR)]). During the follow-up period, 15 [7 to 31] sputum culture tests (median [IQR]) were performed in each patient. Serum anti-GPL-core IgA antibody was positive in 699 patients (71.6%), among which 668 patients subsequently satisfied the ATS/ERS/ESCMID/IDSA guideline diagnostic criteria and were diagnosed with MAC-PD or MAB-PD. The time required for the second positive culture was a median of 51 (IQR 12 to 196) days, suggesting the time-saving effect of adding single culture isolation plus anti-GPL-core IgA antibody as diagnostic criteria. The serum anti-GPL-core IgA antibody was negative in 277 patients (29.4%), among which 223 patients were diagnosed with MAC-PD or MAB-PD. In our cohort, 13 patients who were anti-GPL-core IgA antibody positive and five patients who were anti-GPL-core IgA antibody negative were clinically diagnosed with NTM-PD and treated with clarithromycin (CLR)-containing multidrug therapy, although their sputum culture test was positive only once. We categorized such patients as “probable MAC-PD or MAB-PD”. Definite and probable MAC-PD or MAB-PD accounted for 97.4% of the anti-GPL-core IgA antibody-positive group, which showed that the positive predictive value (PPV) of anti-GPL-core IgA antibody test among patients with radiologically suspected MAC-PD or MAB-PD who have already had single positive sputum culture test was 97.4% in our cohort (Table 2 and Table S1).

**TABLE 1:** Baseline patient characteristics^*a*^

Characteristic	Total (N = 976)	MAC-positive (N = 930)	MAB-positive (N = 46)
Sex, male/female	239/737	227/703	12/34
Age, mean±SD, yr Male Female	68.2 ± 10.9 70.5 ± 10.2 67.4 ± 11	68.3 ± 10.9 70.8 ± 10 67.5 ± 11	67 ± 10.7 65.1 ± 11.4 67.7 ± 10.4
Observation period, median (IQR), d	1518 (867-2594)	1526 (877-2609)	1172 (626-1993)
No. of sputum culture tests, median (IQR)	15 (7-31)	15 (7-32)	13 (8-22)

a MAC, Mycobacterium avium complex; MAB, Mycobacterium abscessus, and its subspecies *abscessus*, subsp. *massiliense*, and subsp. *bolletii*; SD, standard deviation; IQR, interquartile range.

**TABLE 2:** Diagnostic accuracy of single isolation plus anti-glycopeptidolipid-core IgA antibody based on ATS/ERS/ESCMID/IDSA criteria^*a*^

Category	Sensitivity	Specificity	PPV	NPV
All (*n* = 976)				
Definite	74.9 (72.0–77.7)	63.5 (52.9–73.0)	95.6 (93.8–96.9)	19.5 (15.3–24.6)
Definite/probable	74.9 (72.0–77.6)	73.1 (61.5–82.3)	97.4 (96.0–98.3)	17.7 (13.6–22.6)
Definite/probable/possible	73.0 (70.1–75.7)	100 (82.4–100)	100 (99.5–100)	6.5 (4.2–10.0)
				
MAC (*n* = 930)				
Definite	75.4 (72.5–78.2)	63.4 (52.6–73.0)	95.5 (93.7–96.8)	20.0 (15.6–25.3)
Definite/probable	75.4 (72.4–78.2)	73.4 (61.5–82.7)	97.5 (96.0–98.4)	18.1 (13.9–23.2)
Definite/probable/possible	73.5 (70.5–76.2)	100 (82.4–100)	100 (99.4–100)	6.9 (4.4–10.7)
				
MAB (*n* = 46)				
Definite	65.1 (50.2–77.6)	66.7 (20.8–93.9)	96.6 (82.8–99.4)	11.8 (3.3–34.3)
Definite/probable	65.1 (50.2–77.6)	66.7 (20.8–93.9)	96.7 (82.8–99.4)	11.8 (3.3–34.3)
Definite/probable/possible	63.0 (48.6–75.5)		100 (88.3–100)	0

a PPV, positive predictive value; NPV, negative predictive value; MAC, Mycobacterium avium complex; MAB, Mycobacterium abscessus, and its subspecies *abscessus*, subsp. *massiliense*, and subsp. *bolletii*. Data are presented as % (95% confidence interval).

Next, we examined 18 patients in the anti-GPL-core IgA antibody-positive group and 49 patients in the anti-GPL-core IgA antibody-negative group, who had been followed up without CLR-containing multidrug therapy because they did not meet the ATS diagnostic criteria (1997) or ATS/IDSA diagnostic criteria (2007) for NTM-PD (16, 17). Notably, cases that were thought to be related to contamination by NTM from the respiratory tract were only observed in the anti-GPL-core IgA antibody-negative group (18 patients). The remaining 18 patients in the anti-GPL-core IgA antibody-positive group and 31 patients in the anti-GPL-core IgA antibody-negative group had pulmonary lesions compatible with NTM or bronchiectasis. They were followed up owing to suspicion of NTM-PD. Therefore, we categorized them as “possible” NTM-PD in this study. The comparison of anti-GPL-core IgA antibody levels among “definite,” “probable,” “possible” MAC-PD or MAB-PD, and the “contamination” group is shown in Fig. S1.

**FIG 2:**
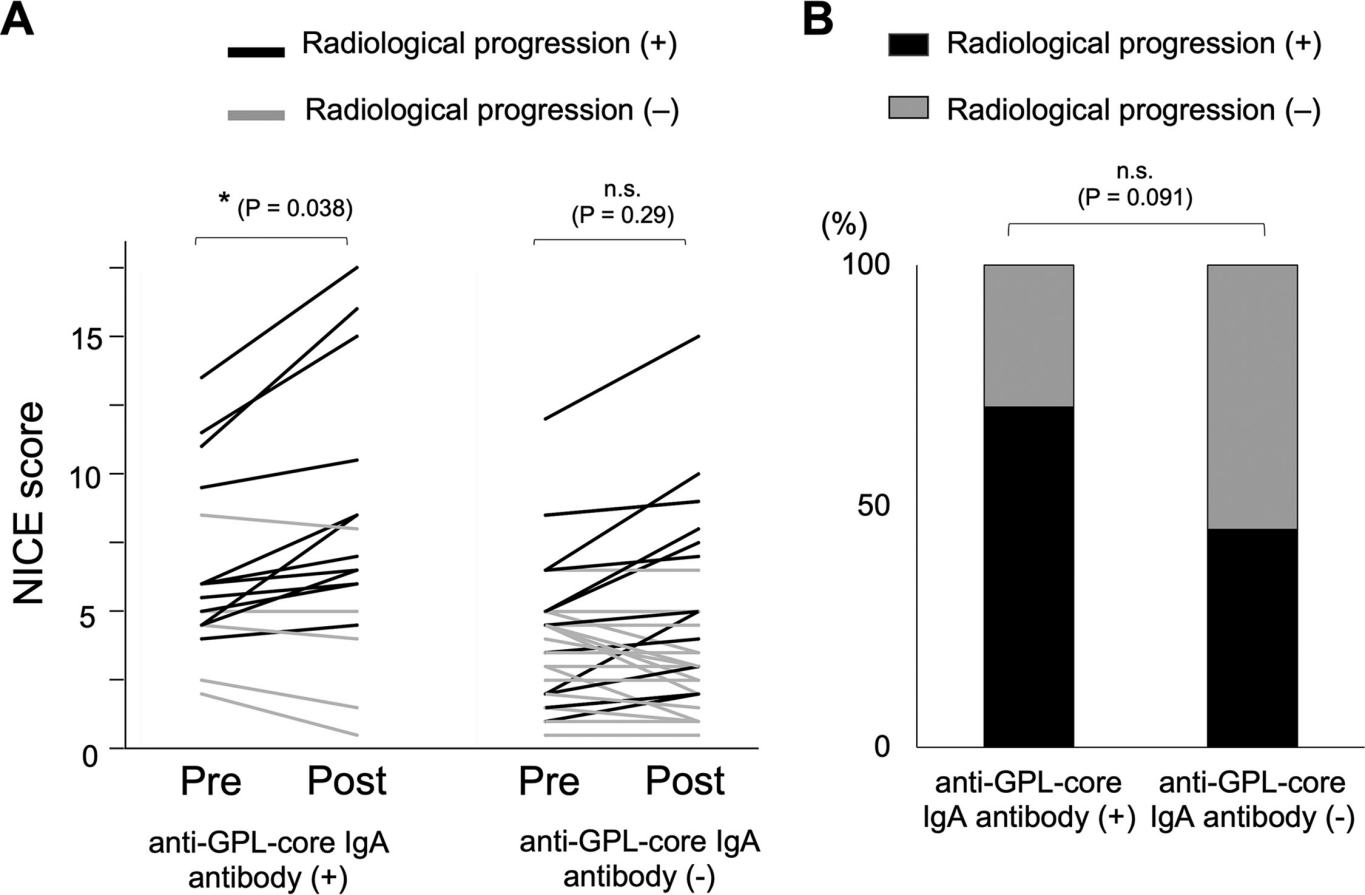
Radiographic evaluation by NICE score among patients with “possible” NTM-PD. (A) NICE (Nodule, Infiltration or consolidation, Cavity, Ectasis) scoring (27) was performed at the first positive sputum culture testing (pre) and after subsequent follow-up (post) among anti-GPL-core IgA antibody positive and negative patients with “possible” MAC-PD or MAB-PD. The comparison between before and after the follow-up period was performed by the Sign test. *, P < 0.05. (B) Percentage of deteriorated (black) and nondeteriorated (gray) patients in anti-GPL-core IgA antibody positive and negative groups were shown. The comparison was performed by a χ^2^ test.

In the “possible” NTM-PD group, we performed radiological estimation by the nodule, infiltration or consolidation, cavity, ectasis (NICE) scoring system at before and after the follow-up period on 17 patients in anti-GPL-core IgA antibody-positive group (one patient in this group lacked radiation image data after the follow-up period) and 31 patients in anti-GPL-core IgA antibody-negative group. The median follow-up period was 46 (IQR 32 to 52.5) months in the anti-GPL-core IgA antibody-positive group and 30 (IQR 17 to 68) months in the anti-GPL-core IgA antibody-negative group and there was no difference between the two groups. The NICE scores before the follow-up period showed a weak correlation with anti-GPL-core IgA antibody levels (Fig. S2A). And the NICE scores in the anti-GPL-core IgA antibody-positive group were significantly higher than those in the anti-GPL-core IgA antibody-negative group before and after the follow-up period (Fig. S2B). During the follow-up period, the NICE scores significantly increased only in the anti-GPL-core IgA antibody-positive group (Fig. 2A). The percentage of patients who showed radiological progression was 70.5% (12/17) in the anti-GPL-core IgA antibody-positive group and 45.2% (14/31) in the anti-GPL-core IgA antibody-negative group, indicating the clinical relevancy of the diagnosis of NTM-PD by the single culture isolation of NTM plus anti-GPL-core IgA antibody among these patients (Fig. 2B).

**FIG 3:**
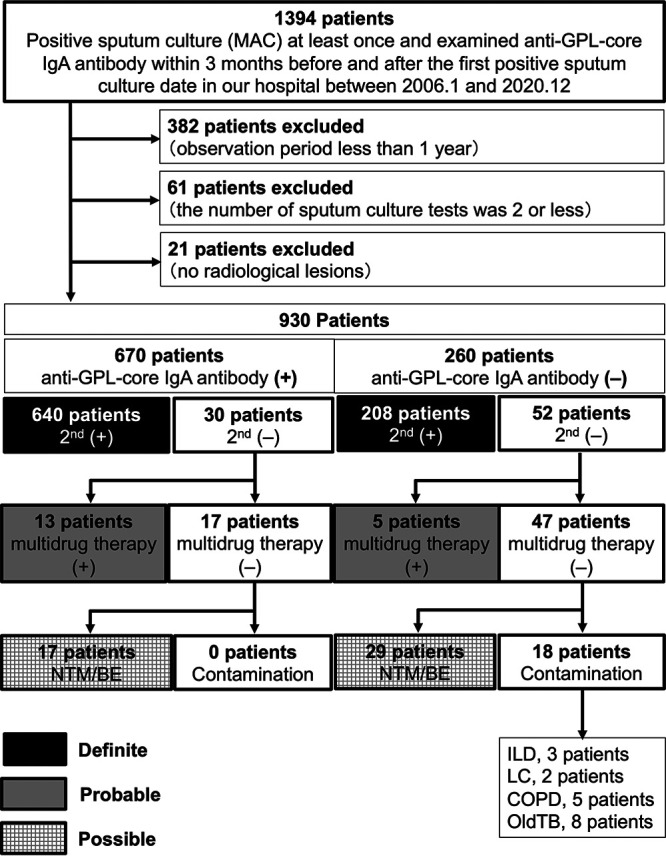
Flowchart of patients with Mycobacterium avium complex pulmonary disease. Anti-GPL-core IgA antibody (+), positive anti-GPL-core IgA antibody (≥0.7 U/mL) at the first positive sputum culture; anti-GPL-core IgA antibody (−), negative anti-GPL-core IgA antibody (<0.7 U/mL) at the first positive sputum culture; 2nd (+), additional positive sputum culture isolation after the first positive sputum culture; 2nd (−), no positive sputum culture isolation after the first positive sputum culture; multidrug therapy (+), presence of history taking multidrug therapy, including clarithromycin; multidrug therapy (−), no history of multidrug therapy, including clarithromycin; NTM/BE, patients with radiological pulmonary lesions compatible with NTM-PD or bronchiectasis; contamination, patients with no obvious radiological pulmonary lesions of NTM-PD or bronchiectasis; ILD, interstitial lung disease; LC, lung cancer; COPD, chronic obstructive pulmonary disease; old TB, past M. tuberculosis disease.

**FIG 4:**
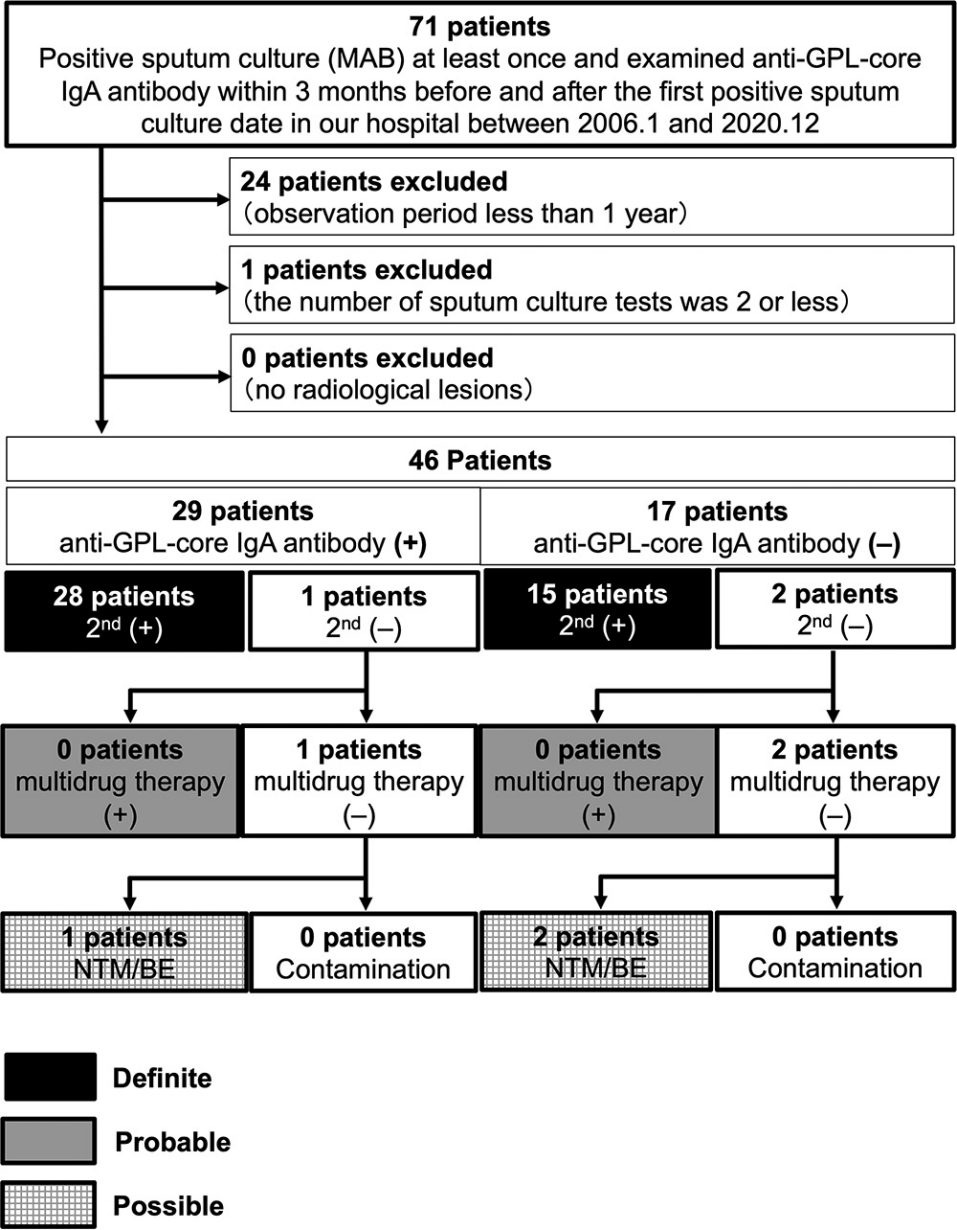
Flowchart of patients with Mycobacterium abscessus pulmonary disease. Anti-GPL-core IgA antibody (+), positive anti-GPL-core IgA antibody (≥0.7 U/mL) at the first positive sputum culture; anti-GPL-core IgA antibody (−), negative anti-GPL-core IgA antibody (<0.7 U/mL) at the first positive sputum culture; 2nd (+), additional positive sputum culture isolation after the first positive sputum culture; 2nd (−), no positive sputum culture isolation after the first positive sputum culture; multidrug therapy (+), presence of history taking multidrug therapy, including clarithromycin; multidrug therapy (−), no history of multidrug therapy, including clarithromycin; NTM/BE, patients with radiological pulmonary lesions compatible with NTM-PD or bronchiectasis; contamination, patients with no obvious radiological pulmonary lesions of NTM-PD or bronchiectasis.

We also performed subgroup analysis limited to MAC-PD (Fig. 3, Table 1, Table 2, and Table S1) and found that the PPV of anti-GPL-core IgA antibody test among patients with radiologically suspected MAC-PD who already have single positive sputum culture test was 97.5%. Similarly, for MAB-PD cases, the PPV was 96.7% (Fig. 4, Table 1, Table 2, and Table S1).

## DISCUSSION

In this study, the PPV of anti-GPL-core IgA antibody test among patients with radiologically suspected MAC-PD or MAB-PD who have already had single positive culture test was 97.4%, which was as high as that obtained by ≥2 positive sputum cultures, the current diagnostic microbiological criteria for NTM-PD (5, 6). We also showed that the median time required for the second positive sputum culture was 51 days in our cohort, suggesting the time, cost, and effort-saving effect of using single culture isolation plus anti-GPL-core IgA antibody as another diagnostic criterion. Although the specificity of anti-GPL-core IgA antibodies has been reported to be different between countries (7, 10, 12–14), it is still high in many countries. Therefore, we propose adding single culture isolation plus anti-GPL-core IgA antibody as “combined microbiological and serological criteria” to the diagnostic guidelines for MAC-PD and MAB-PD.

NTM infection is initiated by the colonization of bronchiolar epithelial cells (18). Once infected, cellular and humoral immunity of the host forms granulomatous lesions and produces various antibodies against NTM (19, 20). Therefore, the production of anti-GPL-core IgA antibodies might prove the presence of infection and distinguish it from contamination (7). In our study, we found that patients with NTM contamination exclusively, were only present in the anti-GPL-core IgA antibody-negative group, reconfirming previous reports. Therefore, if the anti-GPL-core IgA antibody is positive, patients must be carefully monitored by repeating the sputum culture more intensively or by performing bronchoscopy.

Recently, it was reported that no less than 41% of patients were diagnosed with NTM-PD by a single positive sputum specimen in tertiary referral hospitals in the USA (21). The sensitivity of the microbiologic criteria in the current guidelines (two or more positive sputum cultures of the same species of mycobacteria) might be low. And improved diagnostic criteria are needed (22). In our radiological evaluation of patients with “possible” NTM-PD who only had a single positive sputum culture, more than half of them had a clinically significant disease. Therefore, our combined microbiological and serological criteria might help identify these patients.

Although the specificity of the anti-GPL-core IgA antibody for MAC-PD is excellent, its sensitivity was reported to be approximately 70% in a systematic review of 16 studies (9). In our previous study (7), we discussed several possible explanations for the false-negative results of anti-GPL-core IgA antibody: recently diagnosed disease; very low bacterial load; or diversity of immune responses to GPL-core in individual patients, potentially governed by HLA genes (23). In this regard, the next challenge is to identify anti-GPL-core IgA antibody negative patients with NTM-PD by serological testing. Several AFB-related antibodies that are differentially produced among patients with AFB infectious diseases were reported (20, 24). In our preliminary experiments, one or a combination of these antibodies compensated for the shortage of anti-GPL-core IgA antibodies (unpublished data).

Generally, the early treatment of patients by the early detection of symptoms is the rule for every disease. In the current clinical practice of NTM-PD, a patient meets diagnostic criteria for NTM-PD does not necessarily mean antibiotic treatment is required (6). This is because the current multidrug antimicrobial therapy is not curative. However, NTM comprises approximately 200 species. Therefore, establishing an optimal antimicrobial regimen for each NTM species is not realistic. One solution might be the early detection and prevention of aggravation by increasing host immunity or sputum training. Early detection by genomic tests (3) in combination with serological tests, such as anti-GPL-core IgA antibody, will be the next-generation diagnostic criteria for NTM-PD.

### Limitations.

This was a single referral center study, and the participants were all Japanese. Although the positive rate of anti-GPL-core IgA antibodies among healthy individuals is very low in Japan, the USA, and Taiwan (7, 12, 14), it was reported to be quite high in Thailand (10). The high positive rate of anti-GPL-core IgA antibody among healthy individuals might affect the PPV of single culture isolation plus anti-GPL-core IgA antibody. Originally, NTM-PD showed different characteristics among countries. The distribution of pathogens of NTM-PD and the environmental exposure in daily life also showed regional differences (25, 26). Therefore, the diagnostic guidelines might be individualized for each country.

Second, although Japanese medical professionals are familiar with the commercially available anti-GPL-core IgA antibody test, the accessibility to this test is limited in other countries. Third, there was a problem related to referral filter bias because this study was performed in a single center of a national hospital organization that specializes in respiratory diseases. In our hospital, the repeated examination of sputum cultures is routinely performed, and many sputum tests tend to be performed over a short period. Therefore, a prospective multicenter study is needed to overcome the above limitations.

### Conclusions.

The positive serum anti-GPL-core IgA antibody test had a sufficiently high PPV among patients with radiologically suspected NTM-PD with single culture isolation of MAC or MAB to diagnose patients with NTM-PD. Single culture isolation plus anti-GPL-core IgA antibody should be added as “combined microbiological and serological criteria” in the diagnostic guidelines for MAC-PD and MAB-PD.

## MATERIALS AND METHODS

### Study design and patients.

This retrospective study was approved by the Ethical Review Board of the National Hospital Organization, Osaka Toneyama Medical Center (TNH-R-2020062). The medical records of both inpatients and outpatients from which the sputum M. avium, M. intracellulare, and M. abscessus was cultured for the first time in our hospital between 1 January 2006 and 31 December 2020 were retrospectively reviewed. The inclusion criteria were as follows: measurement of serum anti-GPL-core IgA antibody within the period during the 3 months before and after a positive sputum culture test, observation period greater than 1 year, sputum culture number greater than 2, and evidence of radiological lesions. When serum anti-GPL-core antibody was measured more than one time, we selected the data of the day closest to the first positive sputum culture test. We picked up the key words of a nodule, branched shadow, bronchiectasis, cavity, and nontuberculous mycobacteria, from the radiogram interpretation reports, to exclude patients who had no radiographic findings, which suggested a diagnosis of NTM-PD. In this study, patients were called “definite” MAC-PD or MAB-PD when the diagnostic criteria of the current guideline were finally met (6). Among patients, who had only a single positive sputum culture test and showed clinical features consistent with MAC-PD or MAB-PD, we call them “probable” MAC-PD or MAB-PD if multidrug chemotherapy was administered by the attending physician. We called the patients “possible” MAC-PD or MAB-PD if the patients with suspected MAC-PD or MAB-PD were only followed up. We judged the positive sputum culture of MAC or MAB as contamination by the clinical course of the patient, including radiographic changes and histopathologic findings of the biopsy specimen. The final decision of contamination was done after the discussion between two pulmonologists.

### Sputum examination.

Sputum cultures were assessed for acid-fast bacilli (AFB) using a 2% Ogawa egg medium (Japan BCG, Tokyo, Japan) or mycobacteria growth indicator tubes (Becton, Dickinson, Tokyo, Japan). Nontuberculous mycobacterial species were identified using the AccuProbe (Gen-Probe Inc., San Diego, CA, USA), COBAS Amplicor (Roche Diagnostics, Tokyo, Japan) system, DNA-DNA hybridization (Kyokuto Pharmaceutical Industrial, Tokyo, Japan), or TRCReady MAC (Tosoh Bioscience, Tokyo, Japan).

### Measurement of serum anti-GPL-core IgA antibody.

Serum concentrations of anti-GPL-core IgA antibodies were measured using an enzyme immunoassay kit (Tauns Laboratories, Shizuoka, Japan) according to the manufacturer’s instructions. The cutoff value was defined as 0.7 U/mL following the manufacturer’s package insert stating.

### Radiological evaluation.

The nodule, infiltration or consolidation, cavity, ectasis (NICE) scoring system (27) was used to evaluate the radiographic images of NTM-PD. In brief, bilateral lungs were divided into six zones, and the extent of nodule, infiltration or consolidation, cavity, and ectasis of each zone was scored from 0 to 4 and the sum of the scores was calculated. Two were randomly selected from five pulmonologists (TK, AM, TN, TKuge, HK) for each patient. These two pulmonologists independently evaluated chest X-ray or computed tomography (CT) around the first isolation of NTM and those taken most recently. Any discrepancies between the two pulmonologists were resolved by discussion.

### Statistical analysis.

Statistical analyses were performed using JMP Pro 13 (SAS Institute Inc., Cary, NC). Continuous variables were reported as the median and interquartile range or mean and standard deviation. Normal distribution was analyzed by using the Shapiro-Wilk test. The sensitivity, specificity, positive predictive value (PPV), and negative predictive value (NPV) were calculated using 2 × 2 contingency tables, which compared anti-GPL-core IgA tests (positive or negative) with the diagnosis (definite, probable, and possible NTM-PD). The comparison of NICE scores between before and after the follow-up period was performed by the Sign test. Patient groups were compared by Fisher’s exact test or χ^2^ test for categorical variables. Correlation between two variables was examined by Spearman’s rank correlation coefficient (ρ). The comparison of anti-GPL-core IgA antibody levels between groups was performed by the Mann-Whitney U test or the Steel-Dwass test. A two-sided *P* value <0.05 was considered statistically significant.
