# Supplementary material for: The Diagnosis of Nontuberculous Mycobacterial Pulmonary Disease by Single Bacterial Isolation Plus Anti-GPL-Core IgA Antibody
Source: Microbiol Spectr. 2022 Jan 5;10(1):e01406-21. doi: 10.1128/spectrum.01406-21 (PMC8729764; doi:10.1128/spectrum.01406-21)

Supplemental Table 1. Comparison of anti-GPL-core IgA antibody test with the diagnosis (2×2 contingency table).

All (n = 976)		
	Definite	Others
Anti-GPL-core IgA antibody (+)	668	31
Anti-GPL-core IgA antibody (–)	223	54
Total	891	85
	Definite/Probable	Others
Anti-GPL-core IgA antibody (+)	681	18
Anti-GPL-core IgA antibody (–)	228	49
Total	909	67
	Definite/Probable /Possible	Others
Anti-GPL-core IgA antibody (+)	699	0
Anti-GPL-core IgA antibody (–)	259	18
Total	958	18
MAC (n = 930)		
	Definite	Others
Anti-GPL-core IgA antibody (+)	640	30
Anti-GPL-core IgA antibody (–)	208	52
Total	848	82
	Definite/Probable	Others
Anti-GPL-core IgA antibody (+)	653	17
Anti-GPL-core IgA antibody (–)	213	47
Total	866	64
	Definite/Probable /Possible	Others
Anti-GPL-core IgA antibody (+)	670	0
Anti-GPL-core IgA antibody (–)	242	18
Total	912	18
MAB (n = 46)		

	Definite	Others
Anti-GPL-core IgA antibody (+)	28	1
Anti-GPL-core IgA antibody (–)	15	2
Total	43	3
	Definite/Probable	Others
Anti-GPL-core IgA antibody (+)	28	1
Anti-GPL-core IgA antibody (–)	15	2
Total	43	3
	Definite/Probable /Possible	Others
Anti-GPL-core IgA antibody (+)	29	0
Anti-GPL-core IgA antibody (–)	17	0
Total	46	0

Definition of abbreviations: MAC, *Mycobacterium avium* complex; MAB, *Mycobacterium abscessus* and its subspecies *abscessus*, subsp. *massiliense*, and subsp. *bolletii*.

Supplemental Figure 1. Differences in anti-GPL-core IgA antibody levels among diagnostic categories of NTM-PD.

Anti-GPL-core IgA antibody levels (U/ml) were compared among “Definite”, “Probable”, “Possible” MAC- or MAB-PD and “Contamination”. Data are presented as box plot. Comparison among groups were conducted by the Steel-Dwass test. *$p < 0.05$.

Supplemental Figure 2. Relationship between anti-GPL-core IgA antibody levels and NICE scores.

Correlation between anti-GPL-core IgA antibody levels and NICE scores in patients with “possible MAC- or MAB-PD.” Linear regression line (blue) and 95% confidence interval (shaded area) are shown. ρ, Spearman’s rank correlation coefficients (A). NICE scores in anti-GPL-core IgA antibody-positive and -negative patients with “possible MAC- or MAB-PD.” Data are presented as box plot. Comparison between two groups were conducted by the Mann-Whitney U test (B). *$p < 0.05$.

Supplemental Figure 1

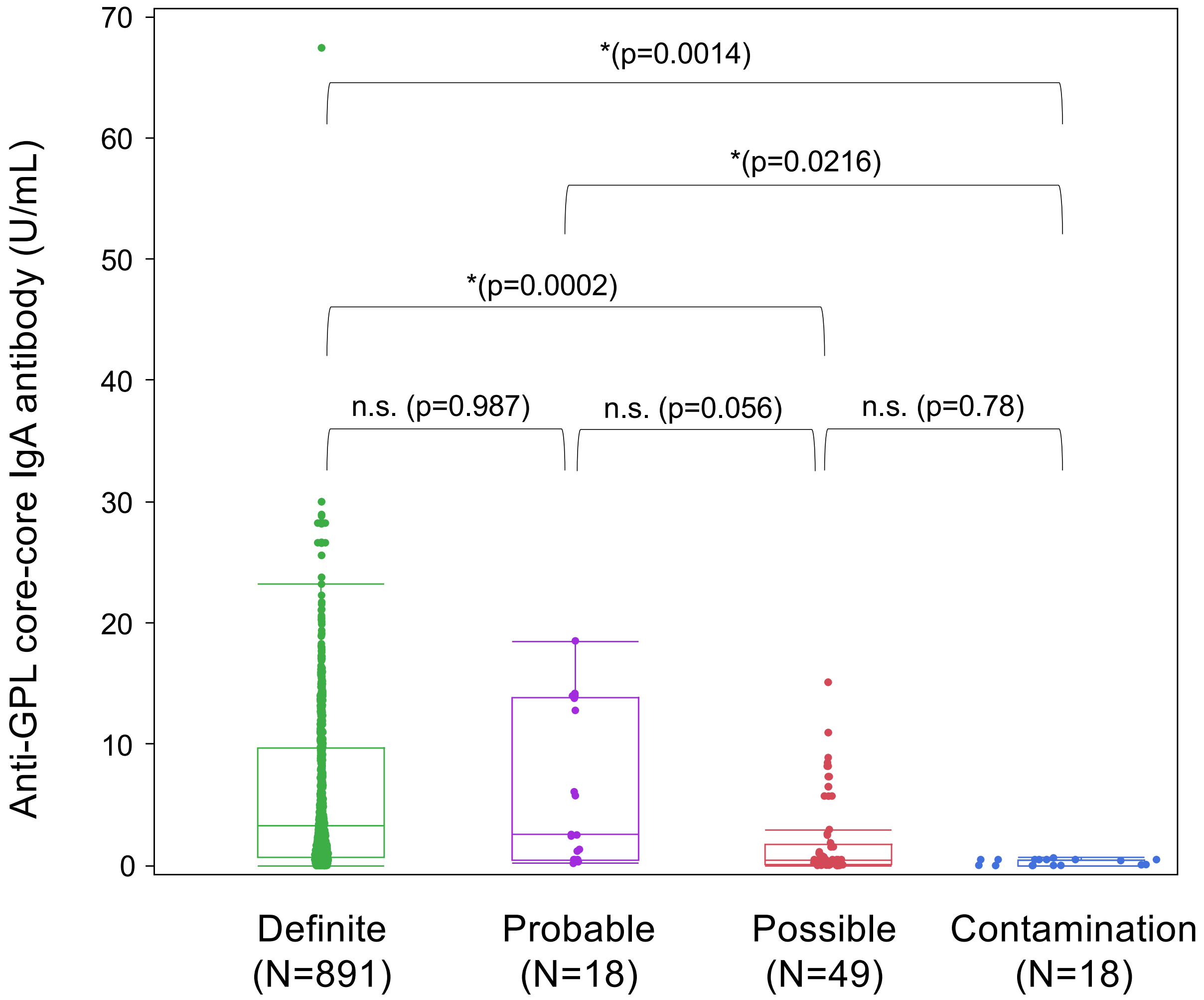

Supplemental Figure 2

A

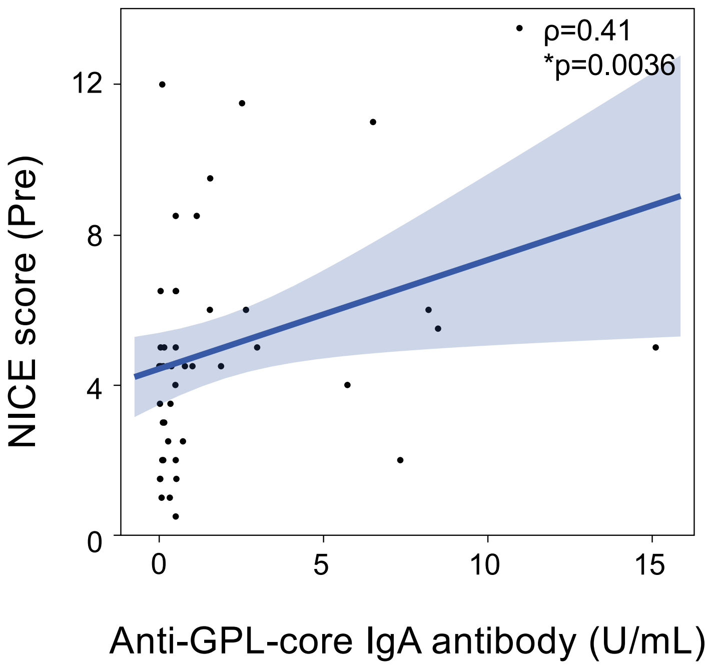

B

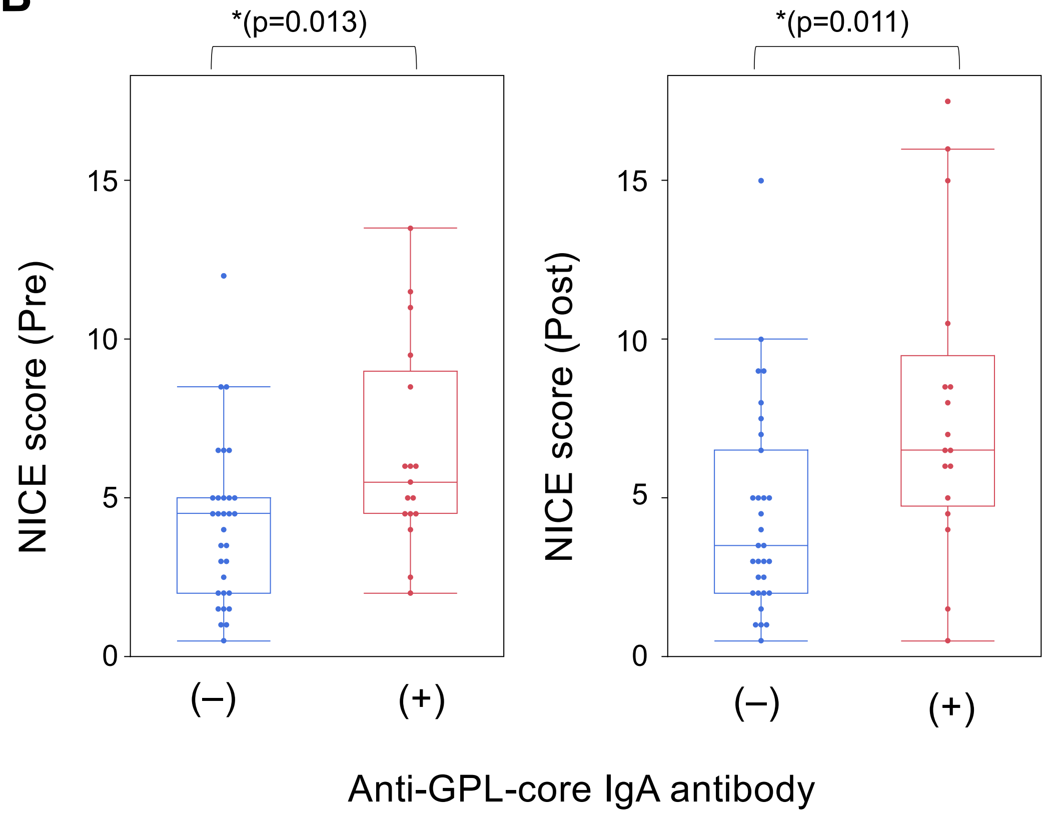

Supplement: SUPPLEMENTAL FILE 1 — Supplemental material. Download SPECTRUM01406-21_Supp_1_seq8.pdf, PDF file, 0.3 MB [file spectrum01406-21_supp_1_seq8.pdf]
